# Electron Spin Resonance Evaluation of Buccal Membrane Fluidity Alterations by Sodium Caprylate and L-Menthol

**DOI:** 10.3390/ijms221910708

**Published:** 2021-10-02

**Authors:** Laxmi Shanthi Chede, Brett A. Wagner, Garry R. Buettner, Maureen D. Donovan

**Affiliations:** 1 Department of Pharmaceutical Sciences and Experimental Therapeutics, College of Pharmacy, The University of Iowa, Iowa City, IA 52242, USA; laxmishanthi-chede@uiowa.edu; 2 Free Radical Radiation Biology, The University of Iowa, Iowa City, IA 52242, USA; brett-wagner@uiowa.edu (B.A.W.); garry-buettner@uiowa.edu (G.R.B.)

**Keywords:** electron spin resonance, membrane fluidity, permeation enhancers, buccal mucosa, sodium caprylate, l-menthol

## Abstract

The ability of sodium caprylate and l-menthol to fluidize phospholipid bilayers composed of lipids simulating the buccal epithelium was investigated using electron spin resonance (ESR) to evaluate the action of these agents as permeation enhancers. 5-Doxyl stearic acid (5-DSA) and 16-doxyl stearic acid (16-DSA) were used as spin labels to identify alterations in membrane fluidity near the polar head groups or inner acyl regions of the lipid bilayer, respectively. The molecular motion of both 5-DSA and 16-DSA showed increased disorder near the polar and inner hydrophobic regions of the bilayer in the presence of sodium caprylate suggesting fluidization in both the regions, which contributes to its permeation enhancing effects. L-menthol decreased the order parameter for 16-DSA, showing membrane fluidization only in the inner acyl regions of the bilayer, which also corresponded to its weaker permeation enhancing effects. The rapid evaluation of changes in fluidity of the bilayer in the presence of potential permeation enhancers using ESR enables improved selection of effective permeation enhancers and enhancer combinations based on their effect on membrane fluidization.

## 1. Introduction

Buccal drug delivery is an alternative to oral and parenteral administration due to the potential for rapid absorption and the lower enzymatic activity present in the buccal tissues compared to the GI lumen, mucosa, and liver [1,2,3]. Several additional advantages for buccal delivery include increased patient compliance due to the convenience and ease of self-application of the dosage form on the inner cheek surface [1,2,3]. However, a significant limitation to the delivery of drugs across the buccal mucosa is the permeability barrier present in the tissue, which limits the systemic absorption of a wide variety of compounds [4,5]. The buccal mucosa is composed of a multilayered, stratified squamous, nonkeratinized epithelium approximately 40–50 cell layers deep with a total buccal tissue thickness of ≈500–800 μm [6,7,8]. Squier et al. demonstrated that the upper one-third to one-quarter of the buccal epithelium acts as the primary permeability barrier to the entrance of drugs and other absorbable materials [9]. The intercellular lipids secreted from the membrane coating granules are major contributors to this permeability barrier [4,9]. These intercellular lipid components have been shown to contain higher quantities of polar phospholipids, cholesterol esters, and glucosylceramides and lower amounts of ceramides compared to the epidermis [10]. Furthermore, histochemical staining has also shown that these polar lipids are highly localized and present as lamellae in the intercellular spaces of the nonkeratinized oral epithelium [10].

The most commonly used approach to modify the buccal mucosal permeability barrier for improved drug absorption has been to incorporate chemical permeation enhancers into formulations [8]. Example categories of materials that have been reported to act as permeation enhancers include surfactants, fatty acids and derivatives, bile salts, chelators, cyclodextrins, positively charged polymers, terpenes, glycols, and short-chain alcohols [11,12,13]. Additional technologies have also been investigated in combination with enhancers to improve buccal drug delivery. A recent study used fused deposition modeling (FDM), a frequently used 3D printing technique, for the preparation of multilayered polyvinyl alcohol (mucoadhesive agent) buccal films with the incorporation of chitosan, which was included as both a permeation enhancer and for its mucoadhesive properties [14]. Results showed a significant increase (≈3-fold) in buccal permeation (tested using porcine buccal mucosa) [14]. In another recent study, a Carbopol 940-based, binary-drug-release buccal film was investigated. It contained a combination of a hydroxylpropyl β-cyclodextrin–simvastatin drug inclusion complex with phosphatidylcholine–sodium deoxycholate mixed micelles. The formulation also contained citral, propylene glycol, and sodium deoxycholate as permeation enhancers. The results showed excellent buccal film properties with enhanced simvastatin permeability across bovine buccal mucosal tissues [15]. Mona et al. also investigated the suitability of mucoadhesive, hybridized nanovesicles loaded with ciclopiroxolamine (antimycotic agent) along with permeation enhancers (Labrafac™ or Labrasol), and their results showed excellent permeation enhancement resulting in superior therapy for buccal candidiasis [16].

Despite their frequent incorporation into buccal delivery systems, the mechanisms by which most enhancers increase the permeation of drugs are not well understood. Many permeation enhancers are known to interact with or alter lipid–lipid associations that facilitate the diffusion of drugs through the perturbed or fluidized microenvironment. It has been widely accepted that buccal permeation enhancement mechanisms can be analogous to their mechanisms of action in the skin, and the mechanisms of skin permeation enhancement have been classified as: (1) affecting or altering the intercellular lipid structure or conformation via extraction or fluidization; (2) altering the conformation of the intracellular proteins, thus increasing intracellular diffusion; or (3) increasing partitioning of the drug or other applied excipients into the stratum corneum [17,18]. Several techniques, including differential scanning calorimetry (DSC) [19], infrared spectroscopy (FTIR) [20], small-angle X-ray diffraction [21], Raman spectroscopy [22], and electron spin resonance (ESR) [23] have been used to investigate the interaction of permeation enhancers with cell membrane components, both in the skin and in the buccal mucosa. These techniques have been used to investigate physical or chemical changes or interactions in membranes following exposure to permeation enhancers in efforts to identify the molecular-level alterations that result in increased barrier permeability.

In previous studies, various permeation enhancers incorporated into buccal film formulations were evaluated for their abilities to improve midazolam permeability across excised buccal tissues [24]. It was observed that L-menthol did not increase midazolam permeation significantly, but sodium caprylate was a very effective permeation enhancer. When both were combined, however, synergistic effects were observed. These unexpected complimentary actions warranted further investigation to determine how the enhancers were interacting with the buccal barrier in order to design improved delivery systems containing single or synergistic combinations of permeation enhancers.

### 1.1. Using ESR to Understand Membrane Fluidity

**Figure 1:**
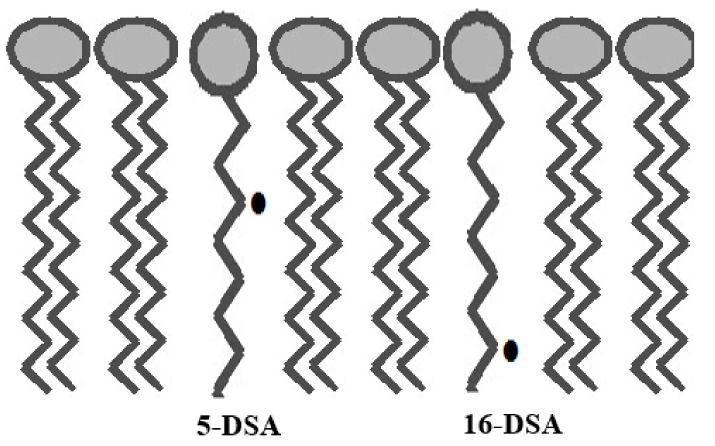
Schematic showing the location of the nitroxide probes, 5-doxyl stearic acid (5-DSA) and 16-doxyl stearic acid (16-DSA), in a phospholipid bilayer. Structures of these two spin probes are presented in Figure 2.

**Figure 2:**
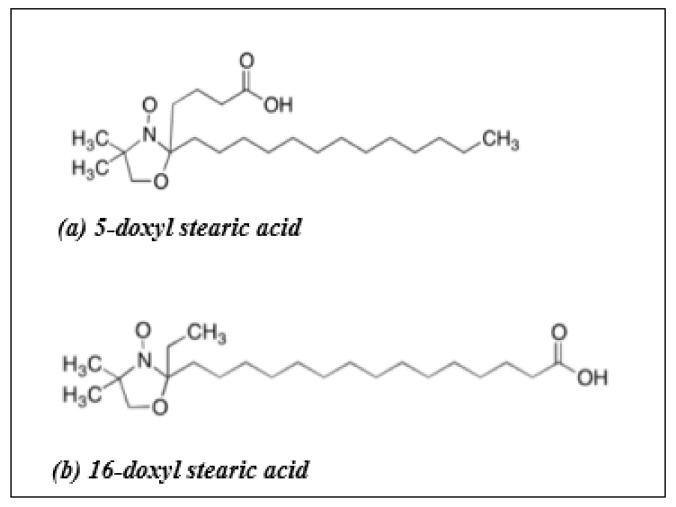
Molecular structures of 5-doxyl stearic acid and 16-doxyl stearic acid [32].

Electron spin resonance (ESR), or electron paramagnetic resonance (EPR), spectroscopy is an important and useful tool to investigate changes in the fluidization of biological membranes. ESR can provide information about the local dynamic properties of the lipid molecules by the incorporation of spin-labels containing a free radical or a paramagnetic center (i.e., a molecule containing at least one unpaired electron) [24,25]. Nitroxide-containing spin-labels are some of the most widely used probes in the study of bilayer fluidity [26,27,28]. Specific lipid (phospholipid) or fatty acid (stearic acid) nitroxide-derivatives have been used to probe the various regions of the bilayer because of the location of a nitroxide in the acyl region of these probe molecules [26,27]. Since these nitroxide spin probes are chemically similar to membrane components (phospholipids, cholesterol), they will localize in the membrane lipid bilayer with their charged carbonyl groups located at the bilayer membrane surface [29,30,31] (Figure 1). In order to investigate changes in bilayer fluidity, specific probes containing nitroxide free radicals (>N-O^•^) on the acyl chain can be chosen which allow placement of the paramagnetic center at different depths within the bilayer in order to monitor the fluidity of the associated lipids [24,25]. In the current study, 5-doxyl stearic acid (5-DSA) and 16-doxyl stearic acid (16-DSA) were used as spin probes (Figure 2). Nitroxide free radical moieties in the 5th (5-DSA) or 16th (16-DSA) carbon position of the alkyl chain will describe the motion profiles in two primary regions of the bilayer, either near the polar head group (5-DSA) or at the end of the hydrophobic chain (16-DSA) (Figure 2) [29,30,31]. Changes in the spin-labels’ motion profile are used to identify conformational change/fluidity in the bilayer and can be related to the ease of diffusion of materials through the perturbed membrane [29,30,31].

Studies on the membrane fluidity of barrier membranes using spin probes have been widely reported in the literature [25,26,27]. The permeation enhancing effect of laurocapram (l-dodecylazacycloheptan-2-one, Azone^®^) on the lipid bilayers of the human stratum corneum has been studied using ESR with 5-DSA as the probe. It was shown that Azone^®^ led to a more fluid environment in the stratum corneum, especially in regions near the polar head groups [26]. Other studies have reported the effects of ethanol and ethanol/l-menthol mixtures on rat stratum corneum using ESR spectroscopy [33]. Three spin labels, androstanol, 5-DSA, and methyl stearate (5-DMS), were investigated, and the ESR results showed that ethanol and l-menthol combinations increased the motion profile of 5-DMS, a probe that localizes in the inner hydrophobic region of the bilayers, demonstrating fluidization of the acyl chains in the phospholipids [33].

### 1.2. Measurement of Membrane Fluidity from ESR Spectra

**Figure 3:**
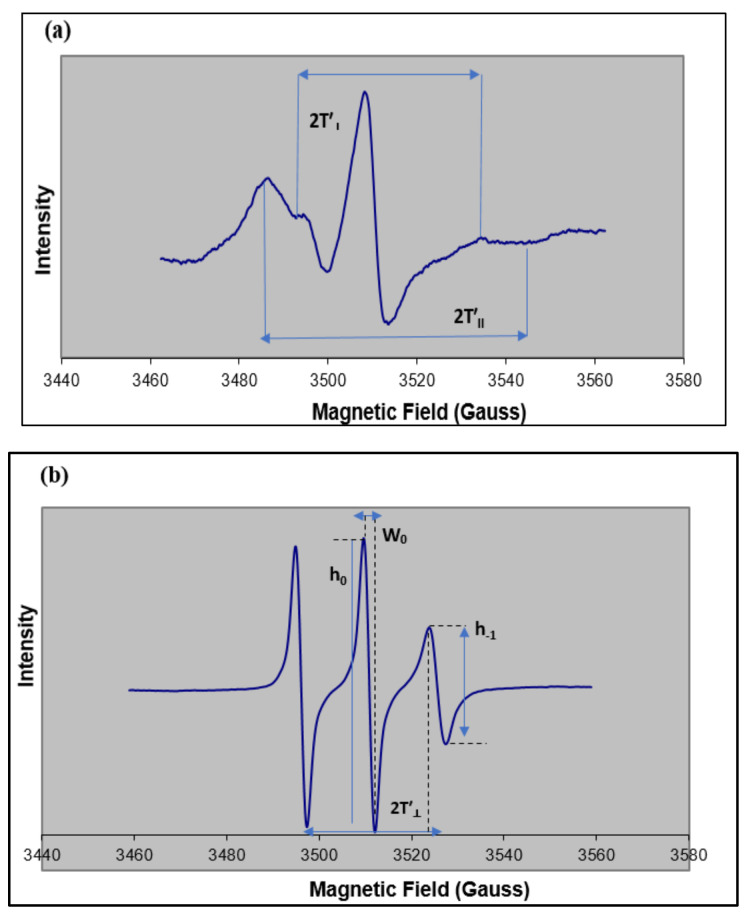
Example ESR spectra showing measurements used to describe membrane fluidity. (**a**) ESR-spectrum of the 5-DSA spin-label incorporated into liposomes (phospholipid:stearic acid:ceramide:cholesterol (1:1:1:1)) measured at room temperature. Shown are the parameters used to determine the order parameter, S. The location of the nadir of the high field offset needed to make the best estimate of 2T’_II_ is best accomplished when the vertical scale of the spectrum is expanded via the Bruker software. (**b**) ESR-spectrum of the 16-DSA spin-label incorporated in chloroform solvent (used as a reference for calculating parameters). Line heights and peak-to-peak line width (W_0_) are used to calculate the rotational correlation time (*τ_c_*). All samples were at room temperature during collection of ESR spectra.

In an ESR spectrometer, a fixed wavelength of microwave radiation is applied to the sample, and the amount of radiation absorbed by the sample (intensity as the dependent variable) is measured when a range of magnetic fields (independent variable) is applied [23,34]. An ESR spectrum (Figure 3) is displayed as the first derivative of the absorption spectrum [23,34]. “Hyperfine splitting” is a unique feature of an ESR spectrum in which the spin interaction with the magnetic nuclei of the sample causes splitting of the first-derivative spectral peaks into smaller components referred to as the hyperfine structure (Figure 3) [35,36]. A compound containing quantum number *I* magnetic nuclei is known to split a single ESR spectral line (peak) into 2*I* + 1 lines [23,35,37]. For example, ^1^H (*I* = 1/2) and ^14^N (*I* = 1) will result in doublet and triplet splitting, respectively [23,35,37]. Nitroxide radicals contain a stable free radical with an unpaired electron that is delocalized between the oxygen and nitrogen atoms [23,35,37]. Based on the 2*I* + 1 rule, a nitrogen radical (*I* = 1) splits into three levels, M_I_ = −1, 0, +1, giving rise to three peaks [30,35] (Figure 3).

The hyperfine interactions that determine the form of an ESR spectrum are described as: (1) anisotropic, which means they depend on the paramagnetic center’s orientation with respect to the magnetic field [34,38], or (2) “isotropic”, which corresponds to no dependence on orientation. Hyperfine splitting occurs when the probe’s movement is restricted, especially in the presence of a viscous solvent or matrix. Two parameters are used to describe the hyperfine splitting observed in an ESR spectrum: T’_II_ (parallel) and T’_⊥_ (perpendicular). These correspond to the hyperfine splittings produced when the magnetic field is parallel to the π orbital axis of the nitroxide bond (T’_II_) or when the field is perpendicular to the π orbital axis (T’_⊥_) [30]. 2T’_II_ is measured as the “outer-peak separation”, and 2T’_⊥_ is defined as the separation between inner hyperfine lines [25,39,40], as shown in Figure 3. An increase in the motion of the probe results in a decrease in parallel hyperfine splitting (T’_II_) and an increase in perpendicular hyperfine splitting (T’_⊥_) [39,40].

#### 1.2.1. Order Parameter (S)

The order parameter is a measure of the angular deviation of the spin probe’s fatty-acid acyl chain at the location of the nitroxide group from the average orientation of the fatty acid chains in the lipid bilayer [25,30,41]. The order parameter S is calculated from the values obtained for the parallel and perpendicular hyperfine splitting (T’_II_, T’_⊥_) [40] using Equation (1).

(1) $$S=1.723\;\left(\frac{T_{\mathrm{II}}^{\prime }-T_{⊥}^{\prime }}{T_{\mathrm{II}}^{\prime }+2T_{⊥}^{\prime }+2C}\right)$$

The correction factor C = 1.4 G − 0.053 (T’_II_ − T’_⊥_); the quantity T’_II_ − T’_⊥_ is defined as the magnitude of the order of the spin probe environment [39,40]. A value of S near 1.0 is characteristic of a more rigid lipid environment [25,39,40], while lower values of S (S < 0.5) are associated with more fluid-like lipid phases [25,39,40].

#### 1.2.2. Rotational Correlation Times (*τ_c_*)

In some instances, when the spin-label motion is isotropic, the order parameter formalism doesn’t apply, and thus is not a reliable indicator of membrane fluidity [42]. Another empirical measure called the rotational correlation time (*τ_c_*) can be used to determine the motion of spin probe when motion tends to be isotropic [39,42]. Rotational correlation times, *τ_c_*, are measured based on Equation (2) [43] where W_0_ is the peak-to-peak line width, in Gauss (G); h_0_ as the line height of the midfield line of the spectrum; and h__1_ as the height of the high-field line (Figure 3).

An ESR spectrum of 16-DSA in a lipid bilayer is often described by the isotropic motion of the acyl chains of both the probe and lipids [39,42], and a decrease in *τ_c_* is associated with an increase in bilayer fluidity [39,42].

*τ_c_* = (6.5 × 10^−10^)W_0_ [(h_0_/h_−1_)^0.5^ − 1] (2)

In the current investigation, sodium caprylate was investigated as a buccal permeation enhancer based on evidence that it is the least cytotoxic, medium-chain fatty acid permeation enhancer [44]. L-menthol is a safe, noncarcinogenic, GRAS-certified excipient currently included in several commercially available buccal films (Orajel^®^, Chloraseptic^®^, Suppress^®^) [45,46], demonstrating its safety in systems applied to the oral mucosa. The combination of sodium caprylate and L-menthol was also investigated based on a previously observed synergism in buccal permeation enhancement of midazolam for this combination [47]. A comparison of sodium caprylate to l-menthol permeation enhancement in those studies showed that 5% sodium caprylate was able to increase the permeability of midazolam through the buccal mucosa to a greater extent than l-menthol when they were included as single agents in buccal film formulations [47].

A multilamellar liposomal system containing equimolar amounts of phosphatidylcholine:stearic acid:ceramide:cholesterol (1:1:1:1), similar to their reported lipid ratios in the buccal tissues, was used as a model buccal lipid membrane to investigate potential bilayer fluidization by sodium caprylate and l-menthol to better understand their mechanisms of buccal permeation enhancement [48].

## 2. Results and Discussion

### 2.1. Effect of Permeation Enhancers on ESR Spectra from 5-DSA

**Figure 4:**
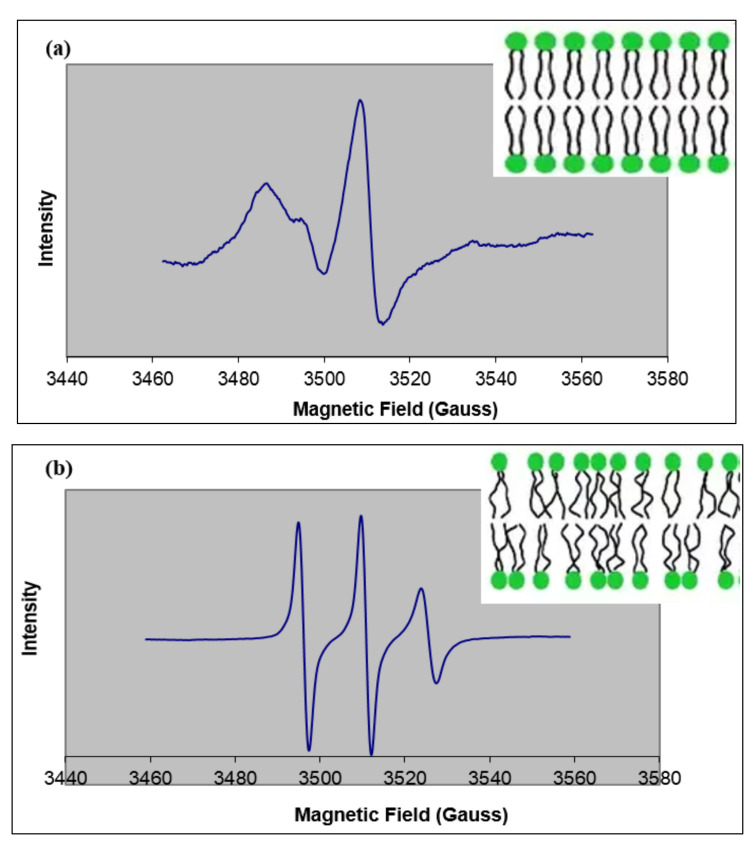
Example ESR spectra from 5-DSA. (**a**) ESR spectrum for 5-DSA incorporated into control buccal prototype liposomes measured at room temperature. Broad spectral lines represent a rigid membrane, as depicted in the insert. (**b**) ESR spectrum for 5-DSA incorporated into liposomes exposed to 5% sodium caprylate and 1% l-menthol measured at room temperature. Narrow spectral lines indicate a fluidized membrane, as depicted in the insert.

Figure 4 shows examples of ESR spectra obtained using 5-DSA and 16-DSA incorporated into buccal-prototype liposomes. The spin parameters obtained when sodium caprylate or l-menthol were also included are reported in Table 1. In 5-DSA, the nitroxide radical is located at the carbon-5 position, and when 5-DSA is intercalated into a bilayer, the paramagnetic center is located near the polar phospholipid head group regions. Changes in spin parameters indicate a change in the organization of the polar region of the bilayer. For control buccal liposomes, an order parameter (S) value of 0.62 indicated a somewhat rigid bilayer. The addition of 5% sodium caprylate and its combination with 1% l-menthol caused the spectral lines to narrow and become more pronounced (Figure 4b). Both of these effects are associated with an increase in the rate of molecular rotation of the spin-label probe molecule around its long molecular axis. The associated changes in the hyperfine splittings (2T’_II_) reduced the S values to 0.42 ± 0.02 and 0.20 ± 0.01 for liposomes treated with 5% sodium caprylate and 5% sodium caprylate+1% l-menthol, respectively (Table 1). In comparison, incorporation of 1% l-menthol showed no change in spectral broadening (2T’_I_, 2T’_II_) compared to control liposomes (Table 1). These results suggest that 5% sodium caprylate alone and in combination with 1% l-menthol can increase the bilayer fluidity in the region close to the polar head groups, but l-menthol failed to generate any change in fluidity in that region.

### 2.2. Effect of Permeation Enhancers on ESR Spectra from 16-DSA

**Figure 5:**
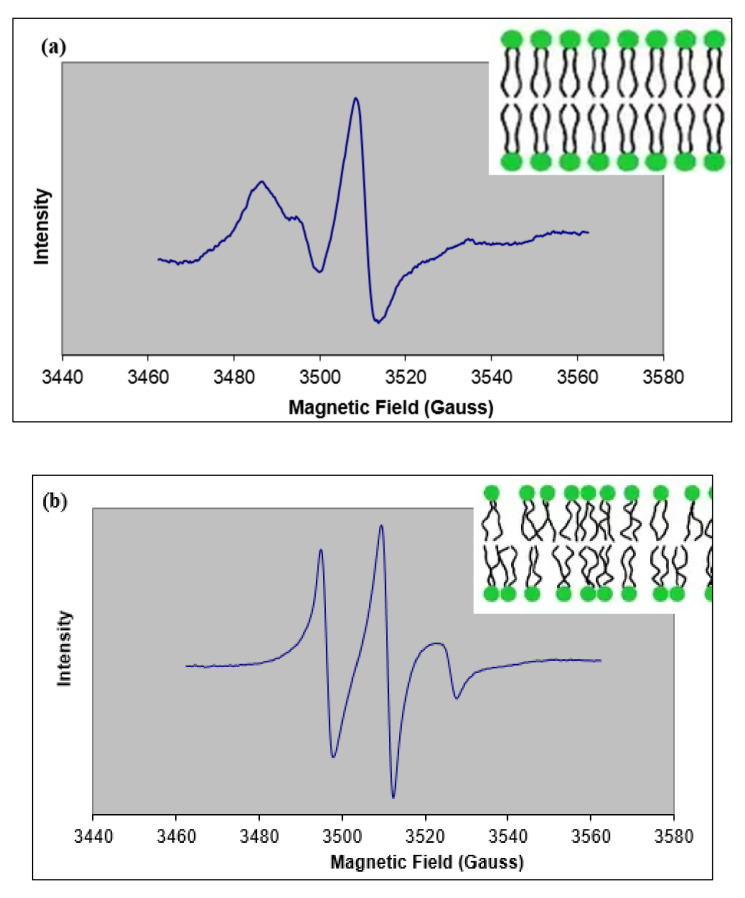
Example ESR spectra from 16-DSA. (**a**) ESR spectrum for 16-DSA incorporated into control buccal prototype liposomes measured at room temperature. Broad spectral lines represent rigid membrane as depicted in the insert. (**b**) ESR spectrum for 16-DSA incorporated into liposomes exposed to 5% sodium caprylate and 1% l-menthol measured at room temperature. Narrow spectral lines indicate a fluidized membrane as depicted in the insert.

In order to study the effect of permeation enhancers on the fluidity of the inner bilayer acyl regions, 16-DSA was used as the spin probe. Figure 5a shows a typical ESR spectrum for 16-DSA incorporated into control liposomes, and Figure 5b shows the ESR spectrum for 16-DSA in liposomes treated with sodium caprylate and menthol. Similar to the 5-DSA results, the inclusion of the permeation enhancers altered the hyperfine splitting compared to the control, and the calculated values of S for all of the formulations containing permeation enhancers showed significant decreases compared to the control liposomes, indicating the fluidity near the C16 position of the phospholipid acyl chain was increased in the presence of both enhancers (Table 2).

Previous investigators have reported that, in the case of 16-DSA, the S value calculation may not be reliable, and the molecular motion of the spin-label is better related to the rotational correlation time, $\tau_{c}$ [25,33]. The $\tau_{c}$ for the control liposomes was 3.4 ns, while formulations treated with sodium caprylate, l-menthol, or a combination of these enhancers showed decreased values for $\tau_{c}$ (Table 2). These lower $\tau_{c}$ values also suggest increased mobility near the ends of the phospholipid acyl chains, and in this case, the results correspond to the observed decreases in S when the permeation enhancers were incorporated into the liposomes.

### 2.3. Comparison of Effects of Permeation Enhancers in Polar and Hydrophobic (Acyl) Regions in a Prototype Buccal Phospholipid Membrane

The spectra observed for 5-DSA and 16-DSA in control liposomes are typical for these probes in a relatively viscous (immobile) state, and several other investigators have reported similar spectra for 5-DSA and 16-DSA in various model lipid bilayer membranes [25,26,39]. Decreases in the order parameter value (S < 0.6) for both probes suggest that bilayer fluidization could be a significant contributor to the observed permeation enhancement activity of sodium caprylate and l-menthol. Other investigators have also reported that sodium caprylate alters lipid bilayer structure, including Sharma et al., who reported that sodium caprylate caused a significant decrease in the fluorescence polarization of the hydrophilic probe, 5-ANS (anilino-1-naphthalene sulfonate), used to label intestinal brush border membrane vesicles. This suggested that sodium caprylate (0.063%, 0.15%, 0.25%) caused membrane disorder in the region close to the polar head groups [49]. In the same study, with an increase in concentration of sodium caprylate to 0.25%, a significant change in the fluorescence polarization of the lipid-soluble probes, DPH (1,6–diphenyl-1,3,5-hexatriene) and 12-AS (anthroxyl–stearic acid), suggested that sodium caprylate may also fluidize the lipid regions near the polar headgroups in addition to the acyl regions of the bilayer at this higher concentration [49]. The authors suggested that this increase in fluidity could be due to either disruption of intermolecular bonds among the tightly packed lipids of the bilayer membrane or to micelle formation and extraction of lipid components from the bilayer.

Evaluation of the fluidization mechanisms of l-menthol on the buccal prototype liposomes showed that there was no significant difference in the S value in the 5-DSA systems, but the S value of the 16-DSA system was significantly decreased. This suggests that menthol partitioned further into the bilayer acyl chain region, causing significant perturbations among the lipid chains. These results are in agreement with previous reports for the stratum corneum and for DPPC vesicles used as bilayer membrane models [25,49]. Additional reports have also shown that the addition of terpene/terpene mixtures to phospholipid liposomes or invasomes significantly increases the vesicle fluidity around the C16 region (using 16-DSA) of the phospholipid acyl chain [25,50]. Narishetty et al. investigated the effect of the fluidization behaviors of terpenes (5% w/v l-menthol, 1,8-cineole) at 37 °C on a model stratum corneum lipid system containing ceramide, palmitic acid, and cholesterol using both DSC and ATR-FTIR. Their DSC results showed that the presence of both terpenes (1,8-cineole and l-menthol) resulted in a very broad, single-transition endotherm with a mid-transition temperature (T_m_) reduced by more than 10 °C compared to the stratum corneum lipid control, indicating a change in lipid phase behavior due to the interactions among the probe and the intercellular lipids. ATR-FTIR studies demonstrated that incorporation of 1,8 cineole and l-menthol with the model stratum corneum lipids generated alterations in CH_2_ stretching vibrations (both symmetric and asymmetric) and amide-I frequencies. Changes in CH_2_ stretching vibrations were indicative of a change/disruption in intercellular lipid chain packing, and the change in amide-I frequencies suggests the terpenes likely disrupted the interlamellar hydrogen bonding network present in the polar head group region [51]. The higher concentration of l-menthol used by Narishetty et al. may be responsible for the observed changes in fluidity near the polar head group region compared to the absence of any increase in polar region fluidization observed using 1% l-menthol in the buccal liposome model. Other studies performed using ESR (24 °C) with 1% of a terpene mixture (cineole:citral:D-limonene- 0.45:0.45:0.1) in 3.3% ethanol showed a significant increase in fluidity (studied using 16-DSA probe) around the C16 position (hydrophobic core) of the phospholipid acyl chains in the DPPC vesicles, similar to the results seen in the current studies [25,48]. The ESR studies investigating model buccal lipid bilayers were conducted at room temperature, and it is likely that the fluidity of these lipids would also be increased at physiologic temperatures, similar to the results reported by Ogiso et al. where the fluidity of stratum corneum lipids was increased with an increase in temperature (25–50 °C) [52]. In their studies, ESR spectra were obtained from stratum corneum lipids using 16-DSA, and the measured rotational correlation times decreased with increasing temperature. At 37 °C, the rotational correlation time was decreased by ≈2 units compared to the value obtained at 25 °C [52]. Sodium caprylate and l-menthol in combination or sodium caprylate alone at ambient temperature were observed to fluidize both the polar and hydrophobic regions of the membrane. However, the intensity/magnitude of fluidization is expected to be increased at physiological temperature with the use of either individual or combination of permeation enhancers. Such increases in the magnitude of fluidization could further increase the permeation enhancing ability of these compounds.

Ogiso et al. also investigated the relationship between the absorption of terolidine across rat stratum corneum and the membrane fluidity measured using 16-DSA at various temperatures (25, 35, 40, and 50 °C) [53]. Results showed that the rotational correlation time (*τ_c_*) decreased with increasing temperature and a strong correlation (r^2^ = 0.94) between fluidity in the stratum corneum lipids (1/*τ_c_*) and the permeability of terolidine was identified. This clear relationship between membrane fluidity and drug permeation across the stratum corneum shows that using ESR to measure changes in membrane fluidity following exposure to drugs or formulation excipients allows for the rapid evaluation and identification of materials able to act as permeation enhancers. The observed synergistic permeation enhancement resulting from the combination of sodium caprylate and l-menthol is now understood to be the result of sodium caprylate’s effects on both the polar and acyl regions of the lipid bilayer, which is then further amplified by l-menthol’s additional fluidization of the acyl chain region. While the biological membranes present in the stratum corneum, buccal epithelium, or other barrier mucosae are far more complex than the simple lipid bilayers used in ESR, the ability to quickly identify changes to the organization of the lipid bilayers using ESR provides a rapid screening tool to identify compounds capable of increasing membrane fluidity, which may further translate to increases in permeability across the barrier upon further optimization of drug and enhancer combinations.

## 3. Materials and Methods

### 3.1. Chemicals

5-Doxyl stearic acid (5-DSA) and 16-doxyl stearic acid (16-DSA) (spin probes) were purchased from Sigma Chemical Co (St. Louis, MO, USA). Soya bean phosphatidylcholine was obtained from Avanti Polar Lipids (Pelham, AL, USA). Ceramide and stearic acid were from Sigma Chemical Co (St. Louis, MO, USA). Chloroform, methanol, and propylene glycol were from Fischer Scientific (Chicago, IL, USA). Sodium caprylate was obtained from Dow Chemical (Midland, MI, USA). Menthol was from Acros Organics (Morris Plains, NJ, USA). Phosphate buffer salts were from Fischer Scientific (Chicago, IL, USA). Phosphate-buffered saline (PBS buffer) pH 7.4 was prepared as per the European Pharmacopoeia 7.0 [25]. Sodium caprylate (25%) and l-menthol (10%) stock solutions were prepared in a 50% *v*/*v* propylene glycol aqueous solvent system.

### 3.2. Preparation of Bilayer Model for Electron Spin Resonance Spectroscopy

Stock solutions (5 mg/mL) of phosphatidyl choline, stearic acid, and cholesterol were prepared in methanol, and a stock solution of 5 mg/mL of ceramide was prepared in chloroform. Spin probes (5-DSA and 16-DSA) at a concentration of 2 mg/mL were prepared in chloroform. From the prepared stock solutions of the lipid components, a 1:1:1:1 mM lipid component mixture was prepared by adding 140 μL, 57 μL, 77 μL, and 121 μL of phospholipid, stearic acid, cholesterol, and ceramide, respectively, to a 10 mL glass test tube. To this lipid mixture, 16 μL (0.04 mM) of 5-DSA or 16-DSA solution was added, resulting in a spin label-to-lipid component molar ratio of 1:25 (Table 3). The mixture was evaporated to dryness using a nitrogen stream to form a thin film on the surface of the tube, and the dried tube was placed in a desiccator for 2 h at room temperature. The film was hydrated for 30 min using 0.5 mL of 7.4 pH phosphate-buffered saline (PBS) at 40 °C (above phase transition temperature for the lipids) in a sonicator (Qsonica, Newtown, CT, USA), followed by vortexing (Scientific Industries, New York, NY, USA) for 10 min. The hydrated lipids were cooled to room temperature.

Stock solutions of sodium caprylate and l-menthol in 50% *v*/*v* propylene glycol were used. The required volume of the permeation enhancers (Table 3) were added to the liposomal dispersions using a pipette. When combinations of permeation enhancers (sodium caprylate plus l-menthol) were used, both were added simultaneously. No permeation enhancer was added to the control sample; the control sample consisted of 300 microliter of 50% *v/v* of propylene glycol. The mixtures were vortexed and examined for vesicle/liposome formation using a polarized light microscope (Leica, Buffalo Grove, IL, USA). The samples showed the presence of ≈200–300 nm liposomes, and, based on previous reports using these same techniques [49,54]. the liposomal bilayers provided excellent systems for the investigation of lipid interactions using ESR. The prepared liposomal dispersion was transferred into an EPR capillary tube to capture ESR spectra.

### 3.3. Electron Spin Resonance Measurements 

Aliquots of the prepared liposomal dispersions were placed into capillary tubes (Hirschmann^®^ melting point tube (100 mm × 0.8 mm × 1 mm) (Sigma–Aldrich, St. Louis, MO, USA) supported in quartz EPR sample tubes (4 mm O.D., Wilmad-LabGlass, Vineland, NJ, USA) in an ER 4119HS cavity. ESR spectra were gathered using a Bruker EMX EPR spectrometer (Bruker BioSpin; Billerica, MA, USA). The modulation frequency, modulation amplitude, and microwave power used were 100 kHz, 0.5 G, and 15 mW, respectively. All instrument parameters were set to maximize signal-to-noise and yet not distort the spectral line shape. Samples were at room temperature during spectral acquisition. The ESR spectrum for each sample was collected over a period of 5 min with a resolution of 1024 points using a scan width of 100 G. Spectral parameter values needed to determine order parameters and rotational correlation times were extracted with the aid of Bruker WIN-EPR software.

## 4. Summary

ESR spectroscopy shows great potential as a method to investigate the mechanisms of action of permeation enhancers. Utilizing ESR, the membrane fluidization actions of both sodium caprylate and l-menthol were identified, and these actions can be used to explain their permeation enhancing effects in the buccal mucosa. Previous investigations showed that buccal permeability was significantly increased by sodium caprylate but not by l-menthol, yet the combination of sodium caprylate and l-menthol acted synergistically to further increase buccal permeability. ESR showed that sodium caprylate increased the fluidity in lipid bilayers, both near the polar headgroups and in the acyl chain regions. Menthol, in comparison, only increased the fluidity of the acyl region, and the lack of effect on the polar headgroup region reduced its ability to act as a permeation enhancer, since a permeant would still need to overcome the barrier represented by the exterior polar region of the bilayer before reaching the reduced fluidity acyl region. When combined with a permeation enhancer able to increase the fluidity of both the polar and acyl regions, however, l-menthol significantly increased the permeability of the buccal mucosa. These results demonstrate that ESR can be used to rapidly evaluate the ability of potential permeation enhancers to fluidize lipid bilayers and can be used as a screening tool to select and optimize individual or combinations of enhancers during initial formulation development activities.

## Figures and Tables

**Table 1:** Parameters derived from ESR spectra for 5-DSA-labeled buccal liposomes exposed to permeation enhancers.

Formulation	2T’_II_ (Gauss)	2T’_⊥_ (Gauss)	Order Parameter (S)
Liposome (control)	70.0 ± 2.5	25 ± 1	0.62 ± 0.02
Liposomes + 5% sodium caprylate	49 ± 1	23.4 ± 0.5	0.42 ± 0.02 *
Liposomes + 1% l-menthol	65.0 ± 1.8	22.7 ± 0.8	0.61 ± 0.01
Liposomes + 5% sodium caprylate + 1% l-menthol	37 ± 1	24.3 ± 0.7	0.20 ± 0.01 *
5-DSA probe in chloroform	37 ± 0.05	26.01 ± 0.02	ND ^a^

^a^ ND, not determined. In this environment, the motion of 5-DSA is essentially isotropic. Thus, the order parameter formalism does not apply [42]. * Asterisk indicates statistically significant differences in the order parameter (S) among the test and control samples. Statistical significance was analyzed by one-way ANOVA followed by Tukey’s multiple comparison tests (*p* < 0.05).

**Table 2:** ESR parameters of 16-DSA labeled prototype liposomes including permeation enhancers.

Formulation	2T’_II_ (Gauss)	2T’_⊥_ (Gauss)	Order Parameter (S)	Correlation Time (Ns)
Liposome (control)	70.1 ± 1.5	18.9 ± 0.5	0.64 ± 0.02	3.1 ± 0.05
Liposomes + 5% sodium caprylate	46.7 ± 1.0	13.3 ± 0.5	0.41 ± 0.02 *	1.46 ± 0.04 *
Liposomes + 1% l-menthol	45.0 ± 1.8	18.7 ± 0.8	0.40 ± 0.01 *	1.51 ± 0.04 *
Liposomes + 5% sodium caprylate +1% l-menthol	40.0 ± 1.0	17.6 ± 0.7	0.21 ± 0.01 *	0.42 ± 0.02 *
16-DSA probe in chloroform	36 ± 0.05	26.0 ± 0.01	ND ^a^	0.34 ± 0.01 *

^a^ ND, not determined. In this environment, the motion of 16-DSA is essentially isotropic. Thus, the order parameter formalism does not apply [42]. * Asterisk indicates statistically significant differences in the order parameter (S) and correlation time among the test and control samples. Statistical significance was analyzed by one-way ANOVA followed by Tukey’s multiple comparison test (*p* < 0.05).

**Table 3:** Composition of buccal prototype liposomes ^a^.

Formulation	Lipid Components					Sodium Caprylate (25% *w*/*v*) in 50% *v/v* Propylene Glycol (μL)	L-Menthol (10% *w*/*v*) in 50% *v*/*v* Propylene Glycol (μL)	5-DSA ^b^ (2 mg/mL) (μL)	16-DSA ^b^ (2 mg/mL) (μL)	PBS
	PC ^b^ (5 mg/mL) (μL)	SA ^b^ (5 mg/mL) (μL)	CH ^b^ (5 mg/mL) (μL)	CE ^b^ (5 mg/mL) (μL)	50% *v*/*v* Propylene Glycol (μL)					
5-DSA liposomes (Control)	140	57	77	121	300	-	-	8		Qs to 1 mL
5-DSA liposomes + 5% sodium caprylate	140	57	77	121		200	-	8		Qs to 1 mL
5-DSA liposomes + 1% L-menthol	140	57	77	121		-	100	8		Qs to 1 mL
5-DSA liposomes + 5% sodium caprylate + 1% L-menthol	140	57	77	121		200	100	8		Qs to 1 mL
16-DSA liposomes (Control)	140	57	77	121	300	-	-		8	Qs to 1 mL
16-DSA liposomes + 5% sodium caprylate	140	57	77	121		200	-		8	Qs to 1 mL
16-DSA liposomes + 1% L-menthol	140	57	77	121		-	100		8	Qs to 1 mL
16-DSA liposomes + 5% sodium caprylate + 1% l-menthol	140	57	77	34		200	100		16	Qs to 1 mL

^a^ Liposomes were composed of phosphatidylcholine, stearic acid, ceramide, and cholesterol (1:1:1:1 mM) containing spin labels for ESR measurements. The molar ratio of spin labels (5-DSA/16-DSA) to total lipids (phosphatidyl choline, ceramide, cholesterol, stearic acid) was 1:25. ^b^ PC, phosphatidyl choline; SA, stearic acid; CH, cholesterol; CE, ceramide; PBS, phosphate-buffered saline; 5-DSA: 5-doxyl stearic acid; 16-DSA, 16-doxyl stearic acid.

## Data Availability

Data available upon request.
